# There is no pulp necrosis or calcific metamorphosis of pulp induced by orthodontic treatment: biological basis

**DOI:** 10.1590/2177-6709.23.4.036-042.oin

**Published:** 2018

**Authors:** Alberto Consolaro, Renata Bianco Consolaro

**Affiliations:** 1 Universidade de São Paulo, Faculdade de Odontologia de Bauru (Bauru/SP, Brazil). Universidade de São Paulo Universidade de São Paulo Faculdade de Odontologia de Bauru BauruSP Brazil; 2 Universidade de São Paulo, Faculdade de Odontologia de Ribeirão Preto, Programa de Pós-graduação em Odontopediatria (Ribeirão Preto/SP, Brazil). Universidade de São Paulo Universidade de São Paulo Faculdade de Odontologia de Ribeirão Preto Programa de Pós-graduação em Odontopediatria Ribeirão PretoSP Brazil; 3 Centro Universitário de Adamantina (Adamantina/SP, Brasil). Centro Universitário de Adamantina AdamantinaSP Brasil

**Keywords:** Dental concussion, Tooth movement, Orthodontics, Pulp necrosis, Calcific metamorphosis of the pulp

## Abstract

To biologically explain why the orthodontic treatment does not induce pulp necrosis and calcific metamorphosis of the pulp, this paper presents explanations based on pulp physiology, microscopy and pathology, and especially the cell and tissue phenomena that characterize the induced tooth movement. The final reflections are as follows: 1) the orthodontic movement does not induce pulp necrosis or calcific metamorphosis of the pulp; 2) there is no literature or experimental and clinical models to demonstrate or minimally evidence pulp alterations induced by orthodontic movement; 3) when pulp necrosis or calcific metamorphosis of the pulp is diagnosed during orthodontic treatment or soon after removal of orthodontic appliances, its etiology should be assigned to concussion dental trauma, rather than to orthodontic treatment; 4) the two pulp disorders that cause tooth discoloration in apparently healthy teeth are the aseptic pulp necrosis and calcific metamorphosis of the pulp, both only induced by dental trauma; 5) the concussion dental trauma still requires many clinical and laboratory studies with pertinent experimental models, to increasingly explain its effects on the periodontal and pulp tissues.

## RESUMO

Para fundamentar biologicamente por que o tratamento ortodôntico não induz necrose pulpar e metamorfose cálcica da polpa, apresentou-se explicações com base na fisiologia, microscopia e patologia pulpar, bem como, e principalmente, nos fenômenos celulares e teciduais que caracterizam a movimentação dentária induzida. As reflexões finais foram: 1) o movimento ortodôntico não induz necrose pulpar ou metamorfose cálcica da polpa; 2) não há literatura e modelos experimentais e clínicos que comprovem ou minimamente evidenciem alterações pulpares induzidas pelo movimento ortodôntico; 3) quando a necrose pulpar ou metamorfose cálcica da polpa for diagnosticada durante o tratamento ortodôntico ou logo após a remoção dos aparelhos ortodônticos, a sua etiologia deve ser atribuída ao traumatismo dentário do tipo concussão, e não ao tratamento ortodôntico; 4) as duas doenças pulpares que levam ao escurecimento coronário em dentes aparentemente hígidos são a necrose pulpar asséptica e a metamorfose cálcica da polpa, ambas induzidas exclusivamente pelo traumatismo dentário; 5) o traumatismo dentário do tipo concussão requer, ainda, muitos estudos clínicos e laboratoriais, com modelos experimentais pertinentes, para fundamentar cada vez mais os seus efeitos sobre os tecidos periodontais e pulpares.

## INTRODUCTION: HOW TO SEARCH FOR WHAT TO READ AND WHO TO LISTEN TO ABOUT THIS SUBJECT?

The search for biological and clinical basis of pulp alterations induced by orthodontic forces is almost always conducted using keywords as “pulp”, “pulp changes”, “pulp pathologies”, “pulp biology”, “Endodontics” and other keywords related to the pulp. Similarly, when asking someone on the “possible changes, opinions, dogmata and beliefs” on the effect of orthodontic treatment on the pulp tissues, it is very common to search for who putatively investigates and researches the pulp biology and diseases - almost always, endodontists. This occurs because, simply put, there seems to be a logical direct relationship with the dental pulp.

However, if we want to find researches to achieve basis and search for specialists to whom we should listen, we should search for those who specifically investigate the periodontal ligament, because the orthodontic movement is an exclusive phenomenon of the periodontal ligament, rather than of the dental pulp. Textbooks of Endodontics and Dental Traumatology rarely discuss the biology of orthodontic movement, which occurs in several texts of Periodontology.

To deeply understand why the dental pulp is not affected by orthodontic movement, there is the need to deepen into periodontal biology and changes, since the pulp does not participate in tooth movement.

## THE ACTIVE ORTHODONTIC FORCES ARE NECESSARILY LIGHT AND MODERATE

**Figure 1:**
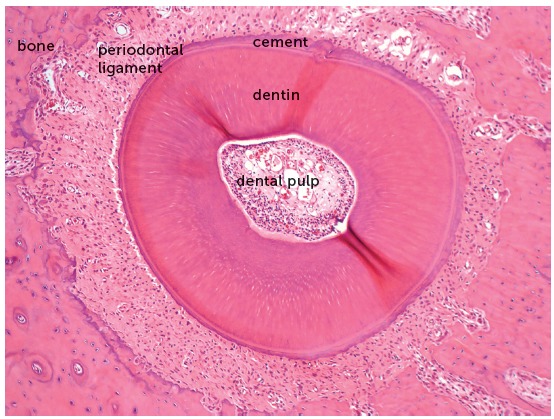
Microscopic aspects of rat molar root in axial or transverse section, revealing the root structures, including the pulp, alveolar bone and periodontal ligament (HE, 10X magnification).

The orthodontic movement is achieved by the application of forces on the tooth, promoting a biological stirring of periodontal ligament cells, known as cellular stress, which may evolve to a mild initial inflammation for some hours or days,^1^ characterized by a mild inflammatory exudate and incipient inflammatory infiltrate (Figs 1 to [Fig f2] [Fig f3] 4). 

**Figure 2 f2:**
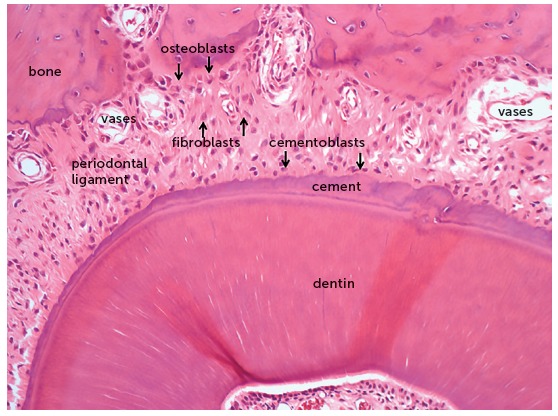
Microscopic aspects, at greater magnification, of the same rat molar root in axial or transverse section of Figure 1, revealing the root structures, including the pulp, alveolar bone and periodontal ligament (HE, 40X magnification).

**Figure 3 f3:**
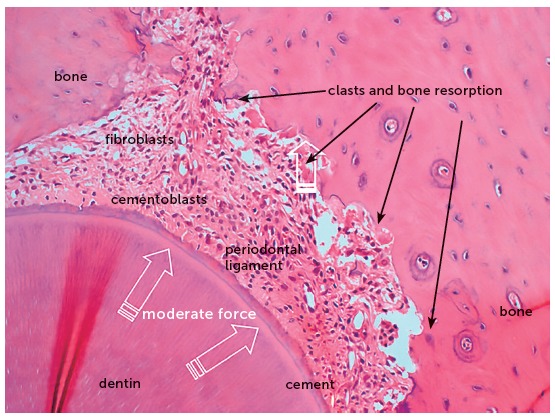
Microscopic aspects of rat molar root, in axial section, after 4 days under moderate orthodontic forces that induced the release of mediators to stimulate the clastic activity on the periodontal bone surface. In this situation, tooth movement occurs slowly, and the forces dissipate without inducing changes in the vascular and neural bundles that penetrate into the pulp via the apical foramen (HE, 25X magnification).

**Figure 4:**
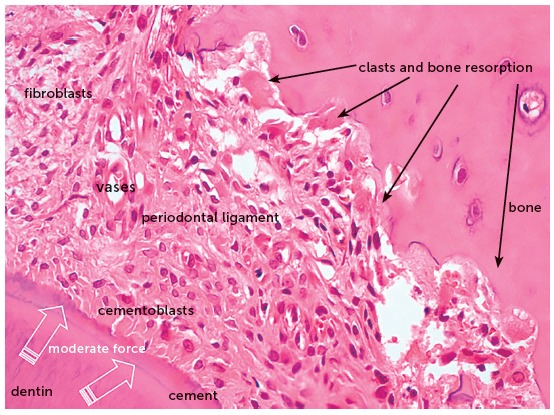
Microscopic aspects of rat molar root presented in Figure 3, at greater magnification, in axial section, after 4 days under moderate orthodontic forces. The image reveals the activity of clasts and other cells, thanks to the maintenance of periodontal structures, without hyalinization (HE, 40X magnification).

The orthodontic forces are very light in any situation, because the goal is to induce these phenomena of cellular stress and periodontal ligament inflammation in the periodontal ligament, which represents a membranous structure of only 0.25mm - which corresponds to the thickness of a paper leaf - formed by specialized fibrous connective tissue.^1^^,^^2^

Besides being very light and moderate, the forces applied on the tooth over the periodontal ligament are dissipating, i.e. they gradually reduce in intensity and disappear in 2 to 7 days, allowing periodontal reorganization, with return to normality between 10 to 15 days after the activation of orthodontic appliances.^1^

Immediately, the orthodontic force applied on the tooth is reduced or partly dissipated by two mechanisms:

1. The liquids and gel represented by the extracellular matrix are displaced to the marrow and perivascular spaces, partially cushioning the effects of the orthodontic forces applied. This represents a physiological mechanism of the periodontal ligament to cushion the heavy masticatory forces.^1^^,^^2^ Considering that this applies to the occlusal loads, which are very intense and incomparably extreme or heavy, by deduction it may be inferred that they apply even more to orthodontic forces, which are extremely light or moderate.

2. Usually, the orthodontic forces applied are reduced in 20 to 30% almost immediately after application, because the alveolar bone crest, which supports the tooth to be moved, undergoes a deflection or deformation due to its elastic or plastic capacity^1^. The alveolar bone tissue is thin and, alike any bone tissue, it presents a high ratio of organic components, liquids and cells.

Over 10 to 12 hours, several clasts or osteoclasts appear on the periodontal bone surface, which initiate the process of bone resorption and enlargement of the periodontal space, which is narrowed by the tooth compression on its structures.^1^ This phenomenon is stimulated by the chemical mediators released by the compressed and hypoxic cells in the periodontal ligament.

After 7 to 10 days, there are nearly no active forces moving the teeth in which the orthodontic forces were applied. After this period, the periodontal phenomena are predominantly reparative, to reorganize the normality of tissues, preparing them to receive another cycle of forces.

Since the first minutes, it is not possible to break the vascular and neural bundles that cross the periodontal ligament and penetrate into the apical foramen to nourish the pulp tissue with blood.^3^ There are no sudden orthodontic movements that might promote partial or total lesions in the blood vessels responsible for the pulp blood supply.

## THE SEVERE OR HEAVY ORTHODONTIC FORCES ARE UNABLE TO MOVE THE TEETH

Since the first moment, the orthodontic forces are dissipating and tend to disappear after 5 to 7 days, in a gradual and decreasing process regarding their intensity.

**Figure 5:**
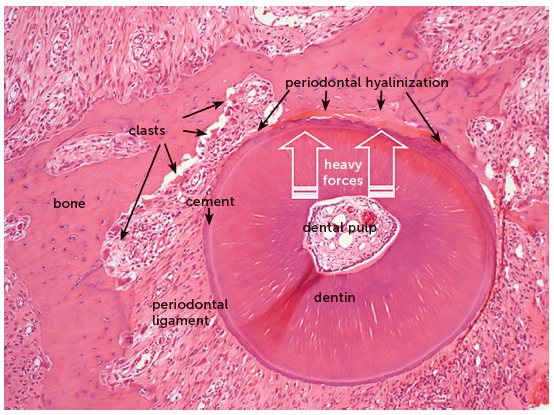
Microscopic aspects of rat molar root presented in axial section after 4 days under heavy orthodontic forces, which induced hyalinization of a segment of periodontal ligament. In this situation there is no tooth movement, because the clasts are unable to resorb the bone on the periodontal surface (HE, 10X magnification).

**Figure 6:**
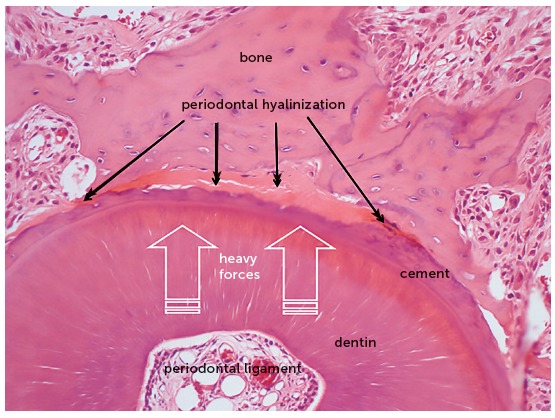
Microscopic aspects of the same rat molar root of Figure 5, at greater magnification, in axial section after 4 days under heavy orthodontic forces, which induced hyalinization of a segment of periodontal ligament. In this situation there is no tooth movement, because the clasts are unable to resorb the bone on the periodontal surface (HE, 40X magnification).

The orthodontic movement of a tooth requires an alive or biologically viable periodontal ligament, that may receive and nourish the clasts that will promote bone resorption on the periodontal surface of the tooth socket (Figs 1 to [Fig f2] [Fig f3] 4). Without vessels with blood supply, without extracellular matrix and mediators, there are no tools necessary for tooth movement in the bone (Figs 5 and 6).

When the forces are very severe or heavy, due to accidental or intentional application of forces that compress the periodontal ligament to an extent enough to occlude the periodontal blood vessels, the cells migrate to neighboring areas to the site of anoxia (Figs 5 and 6). 

The orthodontic movement never occurs suddenly! This sudden characteristic is observed in dental trauma,^4^ not in orthodontic movement. The orthodontic movement and dental trauma promote completely different tissue changes, even though both are caused by forces, which are yet entirely different concerning their intensity, duration and field of action. In the area where heavy forces act on the periodontal ligament, there will remain only the extracellular matrix, without cells nor blood vessels with blood supply, which will be occluded. At this site, the clasts are unable to resorb the alveolar bone and it is impossible to enlarge the periodontal space and achieve tooth movement. Microscopically, these areas that only present extracellular matrix, without cells, are named hyaline areas, or the process is identified as periodontal hyalinization (Figs 5 and 6).

That is to say: if the forces are not light or moderate, there is no tooth movement;^1^^,^^3^ i.e., severe or heavy forces do not allow orthodontic tooth movement. Therefore, if heavy forces are applied by professionals without proper orthodontic training, the risk of pulp necrosis and calcific metamorphosis of the pulp is reduced to zero.

## ORTHODONTIC MOVEMENT AND DENTAL TRAUMA: THEY ARE NOT COMPARABLE

Once again: the orthodontic movement never occurs suddenly! This sudden characteristic is observed in dental trauma, not in orthodontic movement. The orthodontic movement and dental trauma promote completely different tissue changes, even though both are caused by forces, which are yet entirely different concerning their intensity, duration and field of action.

Similarly, since the pulp alterations supposedly assigned to orthodontic treatment are actually related to dental trauma, it is pertinent to investigate the literature or question investigators of dental trauma about this issue.

Dental concussion is the type of dental trauma that may lead to silent aseptic pulp necrosis and calcific metamorphosis of the pulp.^1^^,^^3^^,^^4^ During orthodontic treatment, if there is aseptic pulp necrosis or calcific metamorphosis of the pulp, it may be surely stated that the cause was dental concussion.

The forces of dental trauma induce sudden movements of teeth in the socket, possibly damaging the vascular and neural bundles that penetrate into the apical foramen to nourish the pulp tissues.^4^

As previously mentioned in this paper, the orthodontic forces are dissipating, and the effective occurrence of tooth movement requires light or moderate forces. The intense forces cause hyalinization of the periodontal ligament and do not allow the tissue and cellular phenomena that characterize the tooth movement.

When specialists and investigators of dental trauma are questioned on the periodontal and pulp effects of dental concussion, their responses are usually evasive. The literature about dental concussion and its tissue effects is still underestimated in the field of dental traumatology, which understandably still focuses on experimental models of fracture, luxation, avulsion and replantation.

One reason is the clinical relevance of these most severe types of dental trauma. Another reason is the difficulty to establish clinical and laboratory experimental models to reproduce dental concussion, considering its incipience and subtility.^4^

Dental concussion is the type of dental trauma that does not immediately induce clinical changes after a knock on the affected teeth, and some cases only present mild painful symptomatology or discomfort for some hours, which disappear spontaneously. 

The patient that suffers a concussion does not consciously remind the dental trauma and will rarely report it during an anamnesis after some months or years. The chief complaint of the patient that suffered concussion will only appear after some months or years, with discoloration of the tooth crown.^3^

The coronal discoloration of apparently healthy teeth may only be caused by two pulp pathologies, both induced by the same cause - dental trauma, especially concussion -, namely: aseptic pulp necrosis and calcific metamorphosis of the pulp.^3^^,^^5^^,^^6^

## TWO EXAMPLES OF MORPHOLOGICAL EVIDENCES IN HUMANS AND ANIMALS

In 2000, Valadares Neto^7^ microscopically analyzed the dentin-pulp complex and external root surfaces of human teeth of twelve teenagers, extracted after rapid maxillary expansion, and compared them to the teeth of other three teenagers not submitted to tooth movement. The following could be concluded about the dentin-pulp complex:

1. There was no dentin and pulp alteration, evaluating the immediate response and after 120 days of retention.
2. There was no dentin and pulp alteration, considering two (0.45mm) and four (0.9mm) daily activations of the expanding screw.
3. The rapid maxillary expansion using a modified Haas expander was considered a biologically safe procedure for the dentin-pulp complex.

It should be highlighted that the forces employed in rapid maxillary expansion were sufficiently intense to hyalinize the buccal periodontal ligament and preclude the tooth movement. Despite the severe orthodontic forces applied on the teeth, there were no microscopic pulp alterations.

In 2005, Consolaro^8^ described the tooth movement in 39 rats, with periods of 1 to 7 days, using the model initially developed by Heller and Nanda,^9^ recognized worldwide as the most employed in studies on this subject. The study analyzed pulp tissues,^8^^,^^10^ compared to those of teeth of other 9 animals not submitted to tooth movement. It was concluded that induced tooth movement did not promote morphological alterations in the dental pulp detectable by light microscopy, either degenerative or inflammatory.

Frequently, due to the methodological limitations of studies on the pulp or due to the experimental model employed, studies attempt to detect alterations in molecular oxygenation, as well as biochemical and enzymatic changes, in orthodontically moved pulps, yet the results are not visible and associated with detectable morphological alterations, and even less microscopically.^11^

In nearly all studies on photomicrographs, the dental pulps of orthodontically moved teeth are morphologically normal.

## CONCLUDING REMARKS

1. The orthodontic movement does not induce pulp necrosis or calcific metamorphosis of the pulp.
2. There is no literature nor experimental and clinical models that demonstrate or minimally evidence pulp alterations induced by orthodontic movement.
3. When pulp necrosis or calcific metamorphosis of the pulp is diagnosed during orthodontic treatment or soon after removal of orthodontic appliances, its etiology should be assigned to concussion dental trauma, rather than to orthodontic treatment.
4. The two pulp pathologies that cause coronal discoloration in apparently intact teeth are aseptic pulp necrosis and calcific metamorphosis of the pulp, both exclusively induced by tooth movement.
5. The concussion dental trauma still requires further clinical and laboratory studies using pertinent experimental models, to provide more information on its effects on the periodontal and pulp tissues.
